# The classification of brain network for major depressive disorder patients based on deep graph convolutional neural network

**DOI:** 10.3389/fnhum.2023.1094592

**Published:** 2023-01-26

**Authors:** Manyun Zhu, Yu Quan, Xuan He

**Affiliations:** ^1^College of Medicine and Biological Information Engineering, Northeastern University, Shenyang, China; ^2^Information Center of Shengjing Hospital of China Medical University, Shenyang, China

**Keywords:** deep graph convolutional neural network, major depressive disorder, brain disease classification, functional connectivity, multi-site

## Abstract

**Introduction:**

The early diagnosis of major depressive disorder (MDD) is very important for patients that suffer from severe and irreversible consequences of depression. It has been indicated that functional connectivity (FC) analysis based on functional magnetic resonance imaging (fMRI) data can provide valuable biomarkers for clinical diagnosis. However, previous studies mainly focus on brain disease classification in small sample sizes, which may lead to dramatic divergences in classification accuracy.

**Methods:**

This paper attempts to address this limitation by applying the deep graph convolutional neural network (DGCNN) method on a large multi-site MDD dataset. The resting-state fMRI data are acquired from 830 MDD patients and 771 normal controls (NC) shared by the REST-meta-MDD consortium.

**Results:**

The DGCNN model trained with the binary network after thresholding, identified MDD patients from normal controls and achieved an accuracy of 72.1% with 10-fold cross-validation, which is 12.4%, 9.8%, and 7.6% higher than SVM, RF, and GCN, respectively. Moreover, the process of dataset reading and model training is faster. Therefore, it demonstrates the advantages of the DGCNN model with low time complexity and sound classification performance.

**Discussion:**

Based on a large, multi-site dataset from MDD patients, the results expressed that DGCNN is not an extremely accurate method for MDD diagnosis. However, there is an improvement over previous methods with our goal of better understanding brain function and ultimately providing a biomarker or diagnostic capability for MDD diagnosis.

## Introduction

Major Depressive Disorder is the second cause of disability, with point prevalence exceeding 4% (Ferrari et al., 2013). Currently, the primary diagnosis of MDD is mainly based on the Diagnostic and Statistical Manual of Mental Disorders (Cooper, 2018) or Hamilton Depression Rating Scale (Sharp, 2015). However, the results largely depend on the physicians’ experiences and may sometimes lead to misdiagnoses.

In this context, establishing objective and quantitative biomarkers for identifying MDD may not only provide insights into illness pathophysiology but also promote the development of biologically informed tests for clinical diagnosis and treatment planning. With the development of medical imaging technology, fMRI can be used to reveal the abnormalities of brain functional connectivity of MDD patients. By treating the human brain as a comprehensive network of functionally interacting brain regions and associating it with human behavior, we can make an understanding of how brain tissue changes in psychiatric disorders, which may effectively contribute to MDD diagnosis approaches (He et al., 2017).

In recent years, massive studies have discussed the pathological mechanism of psychosis and tried to search for its biomarkers. Most of them confirm the biological pathology of brain diseases is mostly related to the abnormality of brain FC. Cattarinussi et al. (2022) collected the resting-state fMRI of Bipolar Disorder to study the alteration of the brain functional network between cortical-limbic structures. Park et al. (2022) demonstrated that the severity of clinical symptoms in patients with Generalized Anxiety Disorder had a strong correlation with the strength of FC in brain regions under negative emotional conditions. Hirshfeld-Becker et al. (2019) conducted a pilot study of MDD prediction in Adolescence by intrinsic brain FC. Long et al. (2020) constructed the resting-state brain functional networks with large samples of resting-state fMRI data, indicating that the basis of the neuropathology of MDD is probably related to the abnormal brain FC. Shen et al. (2016) applied resting-state fMRI data to the objective diagnosis of depression patients with or without anxiety by graph theory features and illustrated the importance of resting-state fMRI data for MDD diagnostic research. The brain network FC data constructed from resting-state fMRI data is also a reliable data source for the diagnosis of MDD. As early as 2011, Brier et al. (2011) systematically studied Alzheimer’s disease through the blood oxygen level-dependent resting-state functional connectivity network. Then, Shi et al. (2021) used a progressive three-step machine learning analysis with resting-state FC data to investigate the classification performance of the Machine Learning model in the multi-center large sample dataset. In addition, they applied the Extreme Gradient Boosting model based on resting-state FC data to classify MDD patients and normal controls and evaluated the clinical application value of the data in MDD.

The brain network classification studies are mainly in two streams: traditional machine learning and deep learning. As yet, the most widely used machine learning in the field is the Support Vector Machine (SVM) (Rathore et al., 2017). As early as 2009, Craddock et al. (2009) classified the region of interest (ROI) wise FC of 20 subjects using linear SVM. Currently, the studies in this field increasingly appears. Ichikawa et al. (2020) classified the whole brain FC of 65 patients by logistic regression. Zhu et al. (2021) achieved the classification of 31 subjects by linear SVM. Yan et al. (2021) used SVM to classify the 32 patients’ regional homogeneity. However, there is a dramatic divergence in classification accuracies because of the demographic and clinical heterogeneity across MDD studies. Qin et al. (2022) noted that while there has been an increasing number of publications, the results are inconsistent with their reported classification accuracies varied from 61.7 to 98.4%. In addition, the optimization of machine learning models typically requires adequate training data to mount generalizability across different samples. A large sample size is critical to ensure population-representative model performance and provide reliable information on the biological underpinnings. Substantial previous machine learning studies were performed using single-site datasets with small sample sizes, which leads to huge variability and poor generability in model performance. To eliminate this problem brought by small datasets to the model performance, we choose the large and multi-site dataset for the following studies.

In recent years, the graph convolution network (GCN) is commonly used in classifying psychosis and other fields, such as the optimization of model parameters in the classification of solder paste defects (Sezer and Altan, 2021). It serves as a deep learning model, which is capable of modeling graph data structures like networks and thus fitting the classification of brain networks. Song et al. (2022) achieved the early diagnosis of Alzheimer’s disease on dual-modality fused brain networks by multi-center and multi-channel pooling GCN. They tested on three datasets with sample sizes of 90, 200, and 169, respectively. The results indicate that the method is effective in the early diagnosis of Alzheimer’s disease. However, there is a main drawback for GCN. Zhang et al. (2018) referred that the regular neural networks’ connection structure is based on layers and processing objects are generally tensors combined in a certain way. For disordered images or intricate structures, the GCN has difficulties to identify all features. They proposed a novel neural network architecture–Deep Graph Convolutional Neural Network to extract useful features characterizing the rich information encoded in a graph for classification purposes by a localized graph convolution model. Then, a novel SortPooling layer that sorts graph vertices in a consistent order is designed to train the traditional neural networks on the graphs. The results on benchmark graph classification datasets demonstrated that the proposed architecture achieves highly competitive performance compared to state-of-the-art graph kernels and other graph neural network methods. Moreover, the architecture allows end-to-end gradient-based training with original graphs without the need to firstly transform graphs into vectors. The advantages of DGCNN make it more suitable for dealing with brain networks.

In this study, we complete the classification of the multi-site and large-scale MDD brain resting-state FC networks based on DGCNN. It not only solves the limitation of small samples and the heterogeneousness of the datasets but also discovers a relatively effective classification algorithm for MDD brain networks.

## Materials and methods

### Dataset acquisition

Our study is performed based on 25 datasets from 17 hospitals in the REST-meta-MDD consortium comprising 1,300 MDD patients and 1,128 NCs. They agreed to share the final resting-state fMRI indices of MDD patients and matched NCs from studies approved by local Institutional Review Boards. Consortium members provide basic information, including diagnosis, disease duration, gender, age, education, HAMD-17, and HAMA. All patients are treated under the Diagnostic and Statistical Manual of Mental Disorders IV or the International Classification of Diseases-10. According to these, patients with HAMA score higher than 14 are rated as anxious, and patients with HAMD score higher than 17 are defined as depressed (Yan et al., 2019; Chen et al., 2022). In this study, we excluded data with poor quality following standard quality control procedures in the REST-meta-MDD consortium (Supplementary material and Supplementary Figure 1). Finally, 1,601 participants (830 MDD patients vs. 771 NCs) were included in our analysis. Table 1 details the sample information and disease course in the dataset.

**TABLE 1:** Clinical information table on subjects.

Type	Number	Age	Gender (Male/Female)	Illness (First/Again)	Medical (Yes/No)
MDD	830	34.1 ± 12.4	308/522	522/308	378/452
NC	771	33.1 ± 12.5	318/453	–	–

In this paper, the dataset is the FC correlation results calculated by the predefined seed points after global regression and preprocessed by DPARSF, a MATLAB-and SPM-based resting-state fMRI preprocessing pipeline for the images (Yan and Zang, 2010). The preprocessing operations of Neuroimaging Informatics Technology Initiative files obtained from fMRI including Slice Timing, Realign, Covariates Removed with global signal regression, Spatial Normalization, and Filtering. See Supplementary material for further details.

### The construction of the brain network

The present paper uses Dosenbach’s 160 functional ROIs as a medical template to define the brain network nodes. We then construct functional brain networks using resting-state fMRI data with equal time series between each node. Functional connectivity is calculated by measuring the Pearson correlation coefficient between each of the 160 regions in MDD brain networks (Pearson, 1900).

For each subject, let *x*_*i*_(*t*),*y*_*j*_(*t*) ∈ *R^M^* separately represent the average resting-state fMRI signals for the brain regions i and j at the point t(t=1,2,⋯,T). M and T denote the total number of brain regions and time points, respectively. Then, between the i -th and the j -th ROIs, the correlation γ_*ij*_ can be defined as (1):


(1) $$\gamma_{\text{ij}}=\frac{\sum_{i,j=1}^{\text{n}}\left(x_{\text{i}}-\bar{\text{x}}\right)\,\left(y_{\text{j}}-\bar{\text{y}}\right)}{\sqrt{\sum_{\text{i}=1}^{\text{n}}{\left(x_{\text{i}}-\bar{\text{x}}\right)}^{2}}\,\sqrt{\sum_{\text{j}=1}^{\text{n}}{\left(y_{\text{j}}-\bar{\text{y}}\right)}^{2}}}$$

where $\bar{x}$ and $\bar{y},$ respectively denote the means of the regional resting-state fMRI signals in regions i and j. By computing the Pearson correlation between the average time series for each pair of ROIs, a correlation-based FC network is generated (Furman and Zitikis, 2017; Liang and Xu, 2022) and the symmetric correlation coefficient matrix of 160×160 is shown in Supplementary Figure 2.

**FIGURE 1:**
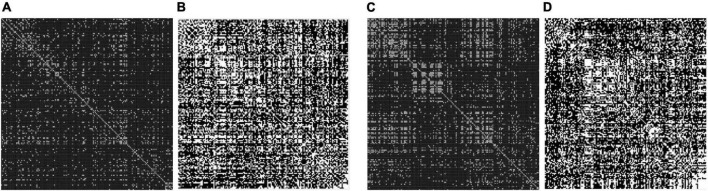
Adjacency matrix with a different threshold on major depressive disorders (MDDs) and normal controls (NCs). **(A)** Threshold = 0.4 MDD adjacency matrix; **(B)** threshold = 0.2 MDD adjacency matrix; **(C)** threshold = 0.4 NC adjacency matrix; **(D)** threshold = 0.2 NC adjacency matrix. The black area indicates no interconnected edges between the two functional brain networks, while the white area indicates connected edges.

To minimize the edge dataset of the brain network, a certain threshold is set to sparse the networks. Then, they are transformed into 0–1 binary adjacency matrices as shown in Figure 1.

**FIGURE 2:**
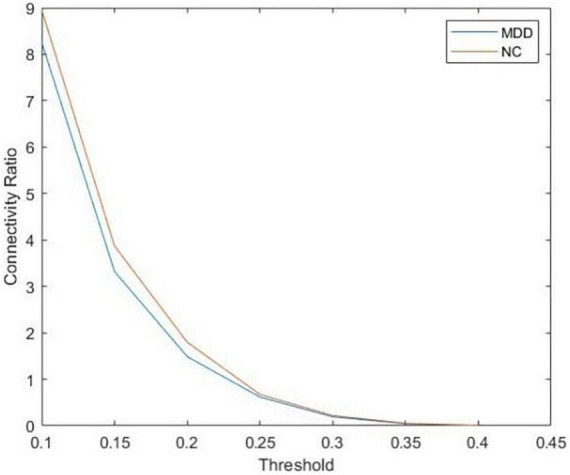
The quantitative ratio curve of brain network connectivity of subjects. The connectivity ratio is the ratio of the number of connected brain networks to the number of unconnected ones for major depressive disorders (MDD) or normal control (NC). With the increase in the threshold, the number of connected graphs dwindled.

After that, we use the Warshall algorithm (Warshall, 1962) to calculate the network connectivity. The n -th order Boolean matrix R(k) (0≤k≤n) expresses whether any pair of nodes in the directed graph contain path information. The quantitative ratio curves of the brain network connectivity of subjects are shown in Figure 2. The Boolean matrix R(k) (0≤k≤n) can be defined as follows:


(2) $${\text{R}}^{k-1}\,\left[i,j\right]+{\text{R}}^{k-1}\,\left[i,k\right]\times {\text{R}}^{k-1}\,\left[k,j\right]\to {\text{R}}^{\text{k}}\,\left[i,j\right]$$

To study the influence of brain network connectivity on the accuracy of MDD final classification prediction, thresholds of 0.2, 0.3, and 0.4 are selected to process the brain network. Connectivity data are then inputted into a deep learning framework. Eventually, we use the DGCNN on connectivity data to identify the full brain network of each subject.

### DGCNN model training

**FIGURE 3:**
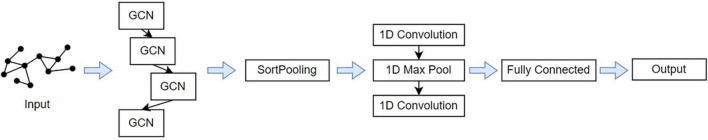
The overall pipeline of deep graph convolutional neural network (DGCNN) classifier distinguishing between individuals with major depressive disorders (MDD) and normal control (NC).

After the brain networks are constructed, a supervised graph classification model is trained by applying the DGCNN algorithm. We aim to extract graph structures of MDD and NC brain networks and available information on nodes. Then, training the model with the presence or absence of disease as the classification label to classify all subjects. The pipeline of the DGCNN model is shown in Figure 3. The model’s input is the graph represented by its adjacency and node features matrices. Individual whole-brain functional connectivity matrices are first represented as graph structures. Nodes are defined as the 160 atlas-based brain regions, and node features reflect the vector of nodal functional connectivity. We get the edges from the binary matrices after setting the different thresholds.

The first four layers are graph convolutional layers. The core process is the spectral graph convolution filter, which can implement the convolution operation on irregular graph data instead of typical data in tensor forms (Kipf and Welling, 2016). The graph convolutional layers with the hyperbolic tangent function activations to improve the convergence speed. The graph’s unordered vertex features from the layers are the input of the next layer, SortPooling, which is a bridge between graph convolution and traditional neural network layers. The SortPooling layer can back-propagate loss gradients, integrating graph representation and learning into one end-to-end architecture. Instead of summing up these vertex features, it arranges them in a consistent order and outputs a sorted graph representation with a fixed size so that traditional convolutional neural networks can read vertices in a consistent order and be trained on this representation.

After SortPooling, a tensor with each row representing a vertex and each column representing a feature channel is generated. To train graph convolutional layers on them, a 1-D convolutional layer is added. Then, Max Pooling layers and 1-D convolutional layers are added to learn local patterns on the node sequence. Two Dense layers are used for binary classification. The convolutional and dense layers use rectified linear unit activation function to introduce non-linearity activation. Finally, the fully connected output layer is activated by a Softmax function to encode output scalars into the predictive probability of each class.

For the parameters of DGCNN, we adopt the default parameters set in the study named “An End-to-End Deep Learning Architecture for Graph Classification” (Zhang et al., 2018). In order to reduce the overfitting, a dropout layer with a ratio of 0.5 is applied between the two dense layers, at which rate dropout generated the most random network structure. The key idea is to drop units randomly (along with their connections) from the neural network during training, which prevents units from co-adapting too much. During the training, dropout layers take samples from the exponential number of different “thin” networks. During the test time, it would be easier to approximate the effect of averaging the predictions of all these thinned networks by simply using a single unthinned network with smaller weights. It significantly reduces overfitting and gives major improvements over other regularization methods. Dropout improves the performance of neural networks on supervised learning tasks (Srivastava et al., 2014). Besides, model training is conducted based on a batch of 50 samples for 100 epochs. The only hyperparameter we need to optimize is the learning rate.

### Description of evaluation indexes for classification performance

To assess the results of each binary classification, we use the following metrics: accuracy (ACC), sensitivity (SEN), specificity (SPE), precision (PRE), F1 score (F1), the receiver operating characteristic curve (ROC), and the area under the ROC curve (AUC) of cross-validation experiment (Bishop and Nasrabadi, 2006). The ROC curve is the plot of the True Positive Rate against the False Positive Rate for different cut-offs of the diagnostic test. It is a measure of the trade-off between sensitivity and specificity. As our analysis has balanced classes (i.e., an equal number of examples for each cognitive state), the ROC-AUC is considered the most important metric (Davis and Goadrich, 2006). The calculation methods are as follows:


(3) $$\text{Accurac}\,y\,\left(\mathrm{ACC}\right)=\frac{\mathrm{TP}+\mathrm{TN}}{\mathrm{TP}+\mathrm{TN}+\mathrm{FP}+\mathrm{FN}}\times 100\%$$

(4) $$\mathrm{Sensitivity}\,\left(\mathrm{SEN}\right)=\frac{\text{TP}}{\mathrm{TP}+\mathrm{FN}}\times 100\%$$

(5) $$\mathrm{Specificity}\,\left(\mathrm{SPE}\right)=\frac{\text{TN}}{\mathrm{TN}+\mathrm{FP}}\times 100\%$$

(6) $$\text{P}\,\mathrm{recision}\,\left(\mathrm{PRE}\right)=\frac{\text{TP}}{\mathrm{TP}+\mathrm{FP}}\times 100\%$$

(7) $$\mathrm{F1}-\text{score}=\frac{2\times \text{PRE}\times \text{SEN}}{\mathrm{PRE}+\mathrm{SEN}}\times 100\%=\frac{2\,T\,P}{2\,T\,P+\mathrm{FP}+\mathrm{FN}}$$

where TP, TN, FP, and FN represent true positives, true negatives, false positives and false negatives of the prediction data.

### DGCNN model function setting

After the dataset with the threshold value of 0.3 is inputted into the model, the loss value and accurate value are calculated. In this paper, the binary cross-entropy loss function is applied. It is commonly used for the two-class classification. The formula is shown in (8):


(8) $$\text{Loss}=-\frac{1}{\text{output}}\,\sum_{\text{i}=1}^{\text{output}}{\text{y}}_{\text{i}}\cdot \text{log}\,\hat{{\text{y}}_{\text{i}}}+\left(1-{\text{y}}_{\text{i}}\right)\cdot \log\,\left(1-\hat{{\text{y}}_{\text{i}}}\right)$$

where i ∈ [1,*output*_*size*], and each i is independent and non-interfering. For this reason, it is suitable for the multi-label classification task. The Adaptive Moment Estimation (Adam) is used as an optimization function, which updates all weights with a constant learning rate alpha during the process. Therefore, dual improvements in quality and speed can be achieved during model optimization (Razak et al., 2021).

## Results

### DGCNN training and prediction results

**FIGURE 4:**
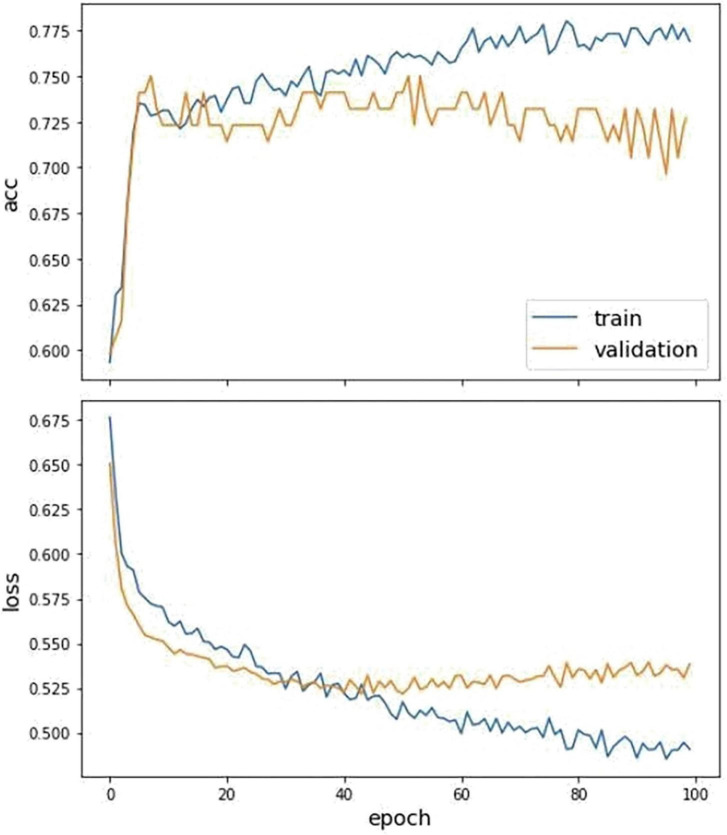
The fitting curve of accuracy and loss of the training and test dataset. The above figure is the training history curve of accuracy, and below is the loss curve. Moreover, the orange is validation, and blue is the training value.

The dataset is split to training and test sets, where 90% of the data is used for training and the remaining 10% for testing. The history of the loss and accuracy of training and testing data are drawn to calculate the performance of the training model based on testing data. The curve is depicted below (Figure 4).

We further make comparisons between DGCNN and other commonly used classifiers, including SVM, Random Forest, and Graph Convolutional Network. For the training of the GCN model, we use the same loss function and optimizer as the DGCNN model to quantify the loss and update the model parameters, respectively. The initial learning rate for the Adam optimizer is set as 0.001. As for the other two classifiers, SVM and RF, a widely used feature reduction strategy, principal component analysis, is adopted before training to avoid over-fitting. SVM and RF are implemented in the Scikit-Learn library with the default setting. Moreover, the parameters of these are optimized using grid search. We use 10-fold cross-validation to examine their performance. The best parameter values set by all methods are shown in Table 2.

**TABLE 2:** The optimal parameters of each method.

Method	Parameters*	Value
GCN	Dropout rate	0.5
	Batch_size	50
	Epoch	100
	Learning rate	0.001
SVM	C	100.0
	Kernel	rbf
	Degree	3
	Gamma	0.01
RF	Max_features	2
	Max_depth	100
	Min_samples_split	200
	Min_samples_leaf	50

*Batch size, the number of data samples captured in a training session; Epoch, the process of training all training samples once; C, penalty term, constraint degree of Lagrange multiplier in SVM; kernel, the parameter for selecting the kernel function in SVM; degree, the degree of the kernel; gamma, the coefficient of kernel function; max_features, the maximum number of features to consider when dividing trees; max_depth, the maximum depth of decision tree; min_samples_split, the minimum number of samples required for internal node partitioning; min_samples_leaf, the minimum sample number of leaf nodes.

The classification results of different methods on this dataset are summarized in Table 3.

**TABLE 3:** Comparison of classification performance indexes of different classification methods in MDD dataset.

Method	ACC* (%)	SPE* (%)	SEN* (%)	PRE* (%)	F1-score (%)	AUC*
SVM (Hearst et al., 1998)	59.7	58.2	61.1	61.2	61.2	0.622
RF (Breiman, 2001)	62.3	60.7	63.7	63.3	63.5	0.647
GCN (Kipf and Welling, 2016)	67.4	66.0	68.7	68.2	68.4	0.701
DGCNN	72.1	67.1	79.2	62.5	69.9	0.788

*ACC, accuracy; SEN, sensitivity; SPE, specificity; PRE, precision; ROC, receiver operating characteristic curve; AUC, area under ROC curve.

It can be seen that our study has superior results. Unsurprisingly, our results are higher than those of traditional machine learning methods. In addition, ours are also higher than the widely used GCN, which demonstrates the effectiveness of the model used in this experiment. Moreover, the fast running speed is also the model’s advantage. Table 4 shows the time required for data loading and classification training at the different thresholds. It indicates that both SVM and RF have a long time running with the worse results than deep learning. The running time of the GCN model is also longer than that of DGCNN, which reflects the superiority of DGCNN on the processing speed of large datasets.

**TABLE 4:** The time cost of dataset loading and training under various thresholds for deep graph convolutional neural network (DGCNN).

Method	Thresholds	Loading time	Training time (epochs = 100)
DGCNN	0.2	214 s	202 s
	0.3	103 s	195 s
	0.4	50 s	191 s
GCN	0.2	724 s	332 s
	0.3	426 s	243 s
	0.4	299 s	220 s
SVM and RF	all	9 h+	8 h+

### Model influencing factors

#### The influence of threshold setting and learning rates on the model

As the number of connected edges and dataset size increase with connectivity ratio, the influence of different thresholds on performance should be discussed. In order to study the relationship between brain network connectivity and classification accuracy, brain networks with thresholds of 0.2, 0.3, and 0.4 are selected for the classification task. Different network connectivity has an impact on classification accuracy. Therefore, we observe the relationship between the classification accuracy and the number of connected edges of the brain network (the connectivity of the brain network) by adjusting the threshold to find the optimal parameter settings and their impact on the optimal prediction result. The size of brain networks with different thresholds is shown in Table 5.

**TABLE 5:** Comparison of brain network sizes with different thresholds.

Thresholds	Nodes	Connectivity ratio	Edge	
			MDD	NC
0.2	160	1.7461	4,200	4,055
0.3		0.2038	2,196	2,020
0.4		0.0037	1,027	917

Undoubtedly, the larger the thresholds, the lower the connectivity index (the number of connected graphs divided by the unconnected graphs) and the smaller the number of connected edges of the brain network.

The comparison of the loss and accuracy values for different thresholds and adjusted learning rate operations is shown in Table 6. It can be seen that the optimal ACC is obtained when the threshold value is 0.3 and the learning rate is 0.001.

**TABLE 6:** Loss and accurate value under different thresholds and learning rates.

Thresholds	Learning rate	Loss	ACC
0.2	0.001	0.5105	0.6852
	0.0001	0.5466	0.6964
0.3	0.001	0.5292	0.7208
	0.0001	0.5367	0.6502
0.4	0.001	0.5419	0.6334
	0.0001	0.5429	0.6384

The above two tables show that the relationship between the accuracy and brain network connectivity or learning rate is non-linear. It means the results will not be significantly affected by extreme connectivity or learning rates and are most optimal under these parameter settings.

The above two tables show that the relationship between the accuracy and brain network connectivity or learning rate is non-linear. It means the results will not be significantly affected by extreme connectivity or learning rates and are most optimal under these parameter settings.

#### The impact of site effect

As the experimental data are obtained from 25 research groups by 17 hospitals in China, the test accuracy is heterogeneous in different research groups and regions. Therefore, the integration of the data into brain network classification for MDD would lead to a large impact on the final result due to data heterogeneity. To eliminate the site effect and the systematic differences around different sites, we conducted leave-one-site-out 10-fold cross-validation experiments on the 16 sites after selection. The classification performance results are summarized in Table 7.

**TABLE 7:** Leave-one-site-out 10-fold cross-validation classification performance.

Site	ACC (%)	SPE (%)	SEN (%)	PRE (%)
S1	67.4	68.4	66.6	74.3
S2	64.1	63.6	64.5	68.3
S7	75.4	78.8	73.0	83.3
S8	72.9	73.0	72.8	76.1
S9	75.1	79.7	72.1	84.7
S10	75.2	74.5	75.9	76.5
S11	84.2	84.4	84.0	86.0
S13	71.1	67.3	75.9	64.9
S14	79.7	73.3	89.1	69.3
S15	61.3	59.9	62.7	62.8
S17	56.9	55.0	58.9	55.9
S19	67.7	64.2	72.1	61.6
S20	70.7	69.9	71.5	72.4
S21	80.8	74.2	90.8	70.1
S22	70.5	71.3	69.9	75.7
S23	69.1	69.4	68.9	73.7

These results demonstrate the reliable classification performance of the model in inter-site cross-validation and applicability of the DGCNN selected in this paper to novel sites. The results combined with cross-validation experiments indicate the advantages of the model on the large-scale multi-site dataset and potential clinical application promotion.

Given the imbalanced single-site performance and sample size across all sites, the current classification performance may be biased by the sites with the best single-site performance and the site with the largest sample size. Besides, it is found that classification accuracy in every single site varied from 56.9 to 84.2%, which confirming the variability of classification tasks with small sample sizes and the importance of using large datasets.

## Discussion

In this paper, artificial intelligence technology is applied to the diagnosis of brain diseases. We use the DGCNN to classify the brain networks of subjects. Systematic experiments are performed on a multi-site and large-scale fMRI dataset collected from the REST-meta-MDD project. Furthermore, we find that when the DGCNN is used as an input feature to the classifier it can provide the best accuracy while classifying subjects into patients and normal controls. It is evident from the experimental results in the previous section, DGCNN has achieved an accuracy of 72.1% on the MDD dataset after ten cross-validations, which is 12.4% higher than SVM, 9.8% higher than RF, and 7.6% higher than GCN. It should be noted that DGCNN also achieves the best AUC and performances in other aspects, which demonstrates the competitiveness of the DGCNN model in this study. Moreover, it requires less time to process large-scale datasets and model training than other models. The rapid classification of 1,601 brain networks can be completed in less than 5 min, which reflects the excellent performance of the DGCNN model in classification speed. Given the high collection cost and small sample size of fMRI data, it is difficult to extract high-dimensional features when training the deep learning model (Khosla et al., 2019). However, generating a large-scale aggregate dataset is expected to solve the problem of repeatability and statistical ability (Borghi and Van Gulick, 2018; Dadi et al., 2019). Therefore, establishing and validating classification models on a large-scale aggregated dataset may promote the development of clinically useful diagnostic methods. In this paper, the results with a large-scale fMRI dataset verify that DGCNN is not very accurate in MDD diagnosis. However, it is improved relatively compared with other machine learning and graph convolution neural networks.

The limitations and future works of this study are as follows. The first problem is that deep learning models may indeed get better results than traditional prediction models, but it is not feasible to identify the important features for prediction. It is a common limitation of the deep learning model. Moreover, only the static FC is used in the classification model, while the potential time-dynamic characteristics are neglected. Further dynamic FC analysis may provide additional useful information for diagnosing brain diseases. Therefore, robust models will be further developed in future research to classify dynamic FC patterns, and related dynamic biomarkers will be explored to diagnose brain disease. On the other hand, this paper uses the Dosenbach template to extract the ROIs in the brain. By comparing with the detailed study of the voxel-level of brain network nodes, it is not sophisticated. Therefore, in future related research, the voxel-level networks will be focal point. Besides the improvement of network construction, it is also worth studying how to enhance model accuracy and further reduce over-fitting. In addition, the impact of issues such as illness duration or the medical status of the prediction results need to be explored as well.

## Conclusion

In this study, we have completed the task of classifying large-scale MDD brain networks based on DGCNN. The overall experimental process is mainly two-fold: brain network construction and classification. During the construction process, the pre-processed dataset of depression fMRI data from the REST-meta-MDD site is used, and the Pearson correlation coefficient matrix is calculated by 160 brain network nodes obtained after the Dosenbach template registration. The obtained correlation coefficient matrix is set to certain thresholds to obtain the 0–1 binary adjacency matrix. The connectivity of the matrix is calculated using the Warshell algorithm. It is concluded that the larger the set threshold, the poorer the connectivity of the brain network. The adjacency matrix is then converted as the input of the DGCNN model. In the classification process, the graph is firstly inputted, and then carry out local extraction. The vertex sorting is executed using the graph convolution layer on the structural characteristics. Then, according to a predefined order by using the SortPooling layer, the model sorts to unify the size of the input graph. Finally, the sorted graph is read with one-dimensional convolution and a dense layer to make the prediction. The model is trained by the specific loss functions and optimization algorithm. Ultimately, DGCNN achieved an accuracy of 72.1% on the large and multi-site MDD dataset after ten cross-validations. It is 12.4% higher than SVM, 9.8% higher than RF, and 7.6% higher than GCN. Moreover, it also has a low time complexity and space complexity. Based on the experimental results, it can be concluded that the DGCNN model has robust performance and fast data processing speed on large-scale datasets. It indicates the model’s effectiveness in brain disease classification and thus provides a promising solution for classification based on fMRI. It further illustrates the potential of deep learning methods in computer-aided medicine.

## Data availability statement

Publicly available datasets were analyzed in this study. This data can be found here: http://rfmri.org/REST-meta-MDD.

## Author contributions

MZ: writing—original draft. YQ: investigation. XH: supervision. All authors contributed to the article and approved the submitted version.
