# Supplementary material for: The classification of brain network for major depressive disorder patients based on deep graph convolutional neural network
Source: Front Hum Neurosci. 2023 Jan 26;17:1094592. doi: 10.3389/fnhum.2023.1094592 (PMC9908753; doi:10.3389/fnhum.2023.1094592)
Supplement: Supplementary file 1 [file Data_Sheet_1.PDF]

Supplementary Material

The classification of brain network for Major Depressive Disorder patients based on Deep Graph Convolutional Neural Network

Manyun Zhu, Yu Quan, Xuan He*

*** Correspondence:** Xuan He: hexuan@bmie.neu.edu.cn

1 Supplementary Data

1.1 Data Selection

To control the data quality and avoid the introduction of bias, we performed a rigorous selection of our data based on the data-selecting procedure of Yan et al. (Yan et al., 2019). Subjects were selected from a total of 2428 subjects (1300 MDDs vs. 1128 NCs) as follows: (1) subjects older than 65 years or younger than 18 years were excluded; (2) subjects with missing information on age, sex, or education year were excluded; (3) low-quality images with bad coverage (< 90% group mask), poor spatial normalization (visual inspection) or excessive head motion (mean framewise displacement (FD) > 0.2) were discarded; (4) to further exclude subjects with distortions that were not detected by visual inspection, we excluded subjects with spatial correlation < 0.6 (a threshold defined by mean - 2SD) between each subject's regional homogeneity (ReHo) map and the group mean ReHo map; (5) sites with subjects fewer than 10 in either MDD or HC group were removed; (6) sites mainly contained patients with geriatric depression subjects were excluded; (7) one duplicated site (Site 4) (detected after consortium data sharing) was discarded. These resulted in 830 MDDs and 771 NCs from 16 sites.

1.2 Data Acquisition, Preprocessing

The preprocessing of fMRI data used in this paper is provided by the DIRECT Consortium of REST-meta-MDD Project (Yan et al., 2019; Chen et al., 2022). A standardized preprocessing pipeline adapted from Data Processing Assistant for Resting-State fMRI (DPARSF) (Yan and Zang, 2010) was implemented at each local participating site to minimize heterogeneity in preprocessing methods. Briefly, it included removing the first 10 volumes for signal equilibrium, slice-timing correction, head motion realignment, brain tissue segmentation, spatial normalization and temporal filtering (0.01-0.10Hz).

a. Covariates: age, gender, educational attainment and head movement

b & c. Motion correction and ICA-based denoising: To control for head motion and physiological noises, the Friston-24 head motion parameters (Friston et al., 1996), liner trend, as well as signals from the white matter, cerebrospinal fluid and whole brain were regressed out. The removal of covariates with global signal regression was finished. Additionally, the mean FD (Jenkinson et al., 2002) was calculated for excluding the subjects with excessive head motion and addressing the residual effects of head motion as a covariate in group analyses.

d. Smoothing level: For better correspondence between symmetric voxels, they further registered the individual functional data to a symmetric template and smoothed (4 mm full width at half maxima).

2 Supplementary Figures and Tables

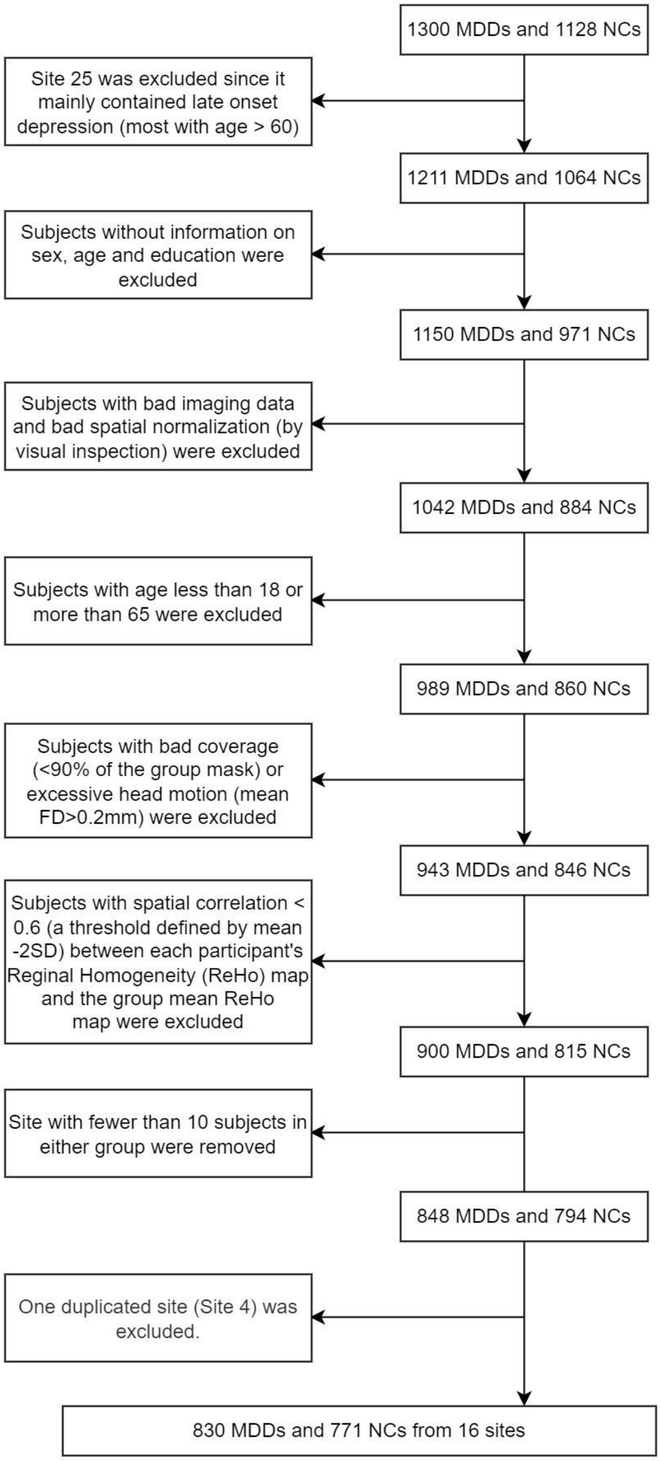

Figure S1. The flowchart of data selection.

Abbreviations: MDD, major depressive disorder; HC, healthy controls; ReHo, regional homogeneity; SD, standard deviation.

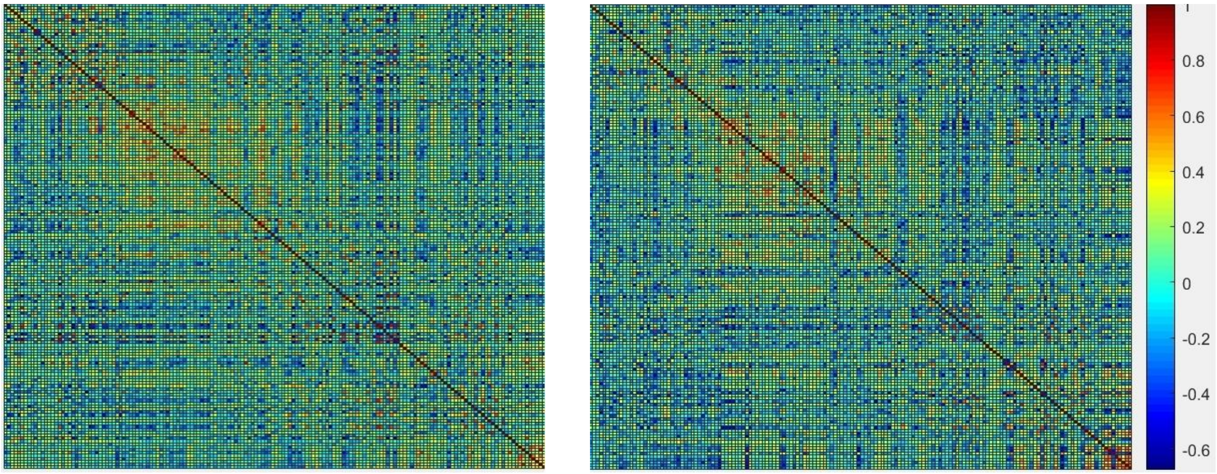

(**a**) (**b**)

Figure S2. Comparison of correlation coefficient matrix of subjects' brain networks. (**a**) The correlation coefficient matrix of MDD; (**b**) the correlation coefficient matrix of NC. The red region shows a strong correlation, while the blue region shows a weak correlation. It can be seen that there is no obvious difference in the correlation matrix of the subjects. However, differences exist among the highest relevance regions.

Reference

1. Yan, C.G.; Chen, X.; Li, L.; Castellanos, F.X.; Bai, T.J.; Bo, Q.J.; Cao, J.; Chen, G.M.; Chen, N.X.; Chen, W. Reduced Default Mode Network Functional Connectivity in Patients with Recurrent Major Depressive Disorder. Proceedings of the National Academy of Sciences 2019, 116 (18), 9078–9083.
2. Chen, X.; Lu, B.; Li, H.X.; Li, X.Y.; Wang, Y.W.; Castellanos, F.X.; Cao, L.P.; Chen, N.X.; Chen, W.; Cheng, Y.Q. The DIRECT Consortium and the REST-Meta-MDD Project: Towards Neuroimaging Biomarkers of Major Depressive Disorder. Psychoradiology 2022, 2 (1), 32–42.
3. Yan, C.; Zang, Y. DPARSF: A MATLAB Toolbox for" Pipeline" Data Analysis of Resting-State FMRI. Frontiers in systems neuroscience 2010, 4, 13.
4. Friston, K.J., Williams, S., Howard, R., Frackowiak, R.S.J., Turner, R., 1996. Movement-related effects in fMRI time-series. Magn. Reson. Med. https://doi.org/10.1002/mrm.
5. Jenkinson M, Bannister P, Brady M, & Smith S (2002) Improved Optimization for the Robust and Accurate Linear Registration and Motion Correction of Brain Images. NeuroImage 17(2):825-841.
